# Mesh removal and reconstruction with posterior components separation technique for delayed mesh infection developed 10 years after abdominal incisional hernia repair: a rare case report

**DOI:** 10.1186/s40792-019-0697-3

**Published:** 2019-09-05

**Authors:** Tetsuro Tamura, Yoshihiro Ohata, Fujio Katsumoto

**Affiliations:** 1 Department of Surgery and Oncology, Graduate School of Medical Sciences, Kyushu University, Fukuoka, Japan; 2 Department of Surgery, JR Kyushu Hospital, Moji, Kitakyushu, Japan; 3 Katsumoto Day Surgery Clinic, Kitakyushu, Japan; 4 Department of Surgery, Shimonoseki City Hospital, 1-13-1 Koyocho, Shimonoseki, Yamaguchi, 750-8520 Japan

**Keywords:** Incisional hernia, delayed mesh infection, components separation technique, MRSA

## Abstract

**Background:**

Very few literatures can be found reporting cases and treatment strategies of late-onset mesh infection after abdominal incisional hernia reconstruction. Here, we report a rare case of delayed mesh infection developed 10 years after abdominal incisional hernia repair, which was successfully treated by mesh removal and reconstruction with posterior components separation technique.

**Case presentation:**

A 66-year-old man, who underwent reconstruction of abdominal incisional hernia by retroperitoneal Composix mesh application 10 years prior, developed 12 × 6.0 × 2.5 cm subcutaneous abscess followed by methicillin-resistant *Staphylococcus aureus* (MRSA)-related mesh infection. The operation was performed excising the abscess wall without damaging peritoneum, and huge intermuscular defect was successfully reconstructed by posterior components separation technique application.

**Conclusions:**

An early decision of excising contaminated mesh would be preferable to extensive conservative treatments when mesh infection is suspected. Components separation technique application can be of great help when designing reconstruction of huge intramuscular defect after removal of infected mesh.

## Background

Incisional hernia is one of the major complications after abdominal surgery. Recently, tension-free reconstruction concept, which is known to reduce recurrence compared to conventional primary closure procedure [1], has widely been accepted for repairing incisional hernia, and therefore, nowadays, most of the reconstructions are performed using mesh. Mesh application is, on the other hand, known to cause complicated infection on occasion. However, very few literatures can be found reporting cases and treatment strategies of late-onset mesh infection after abdominal incisional hernia reconstruction. Here, we report a very rare case of delayed mesh infection developed 10 years after abdominal incisional hernia repair, which was successfully treated by mesh removal and reconstruction with posterior components separation technique.

## Case presentation

**Fig. 1:**
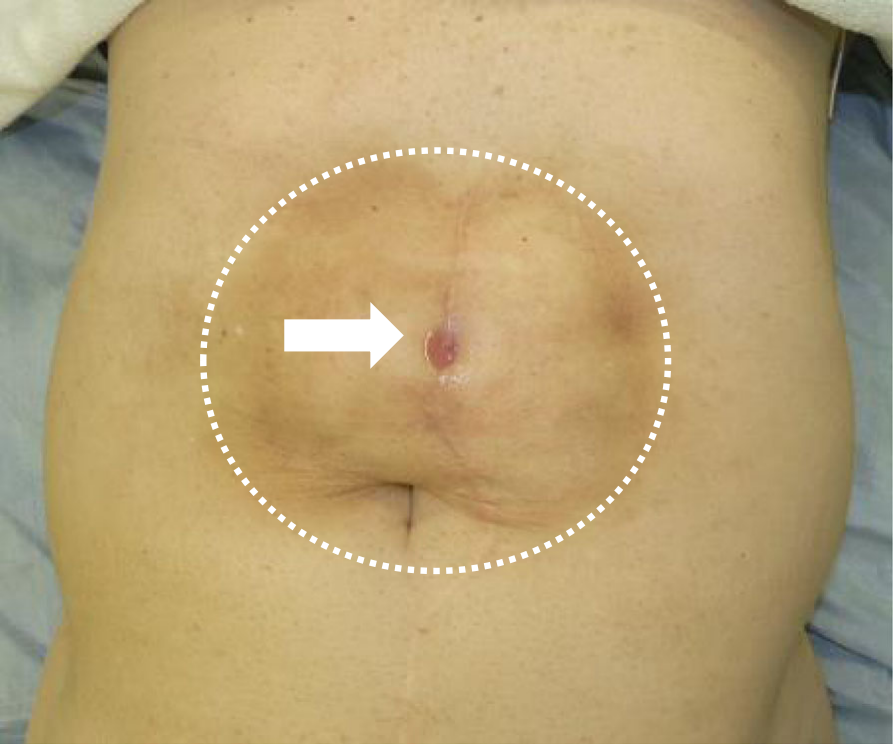
Abdominal appearance of the patient at operation. Dashed circle shows the range of palpable subcutaneous fluid collection and an arrow indicates the skin fistula

**Fig. 2:**
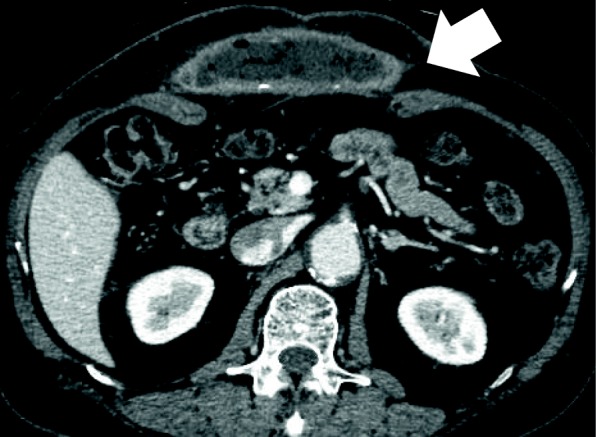
Preoperative CT suggesting subcutaneous encapsulated 12 × 6.0 × 2.5 cm abscess formation (arrow)

**Fig. 3:**
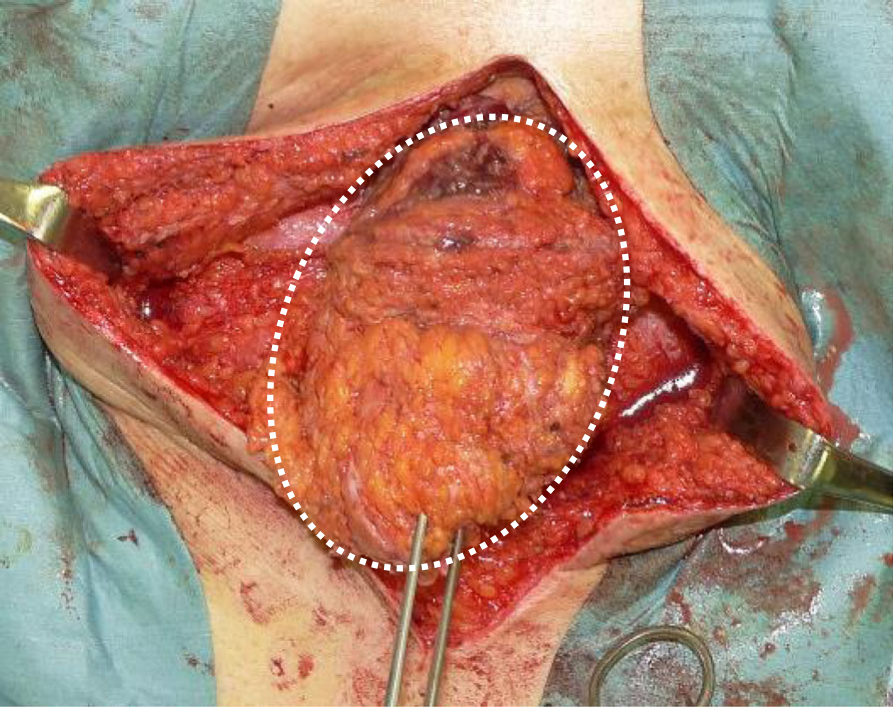
Complete removal of the abscess wall (dashed circle) without damaging the peritoneum and abscess wall was performed

**Fig. 4:**
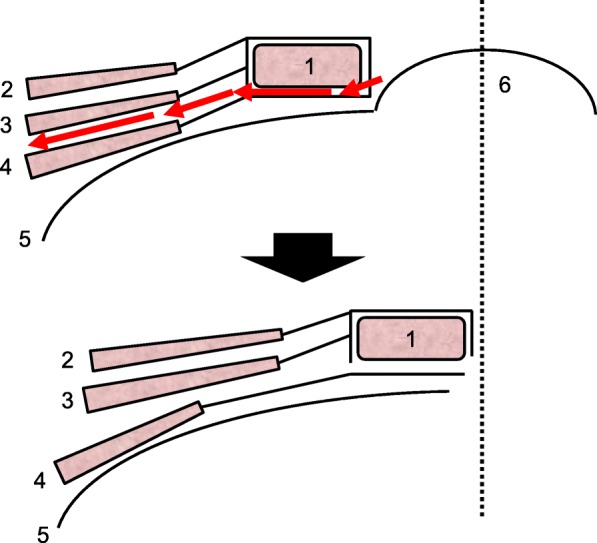
Schema of the posterior components separation technique applied for the present case. Arrows indicate directions of incision. A dashed line shows midline. 1 Rectus abdominis muscle, 2 external oblique muscle, 3 internal oblique muscle, 4 transversus abdominis muscle, 5 transversalis fascia and peritoneum, and 6 hernia sac

**Fig. 5:**
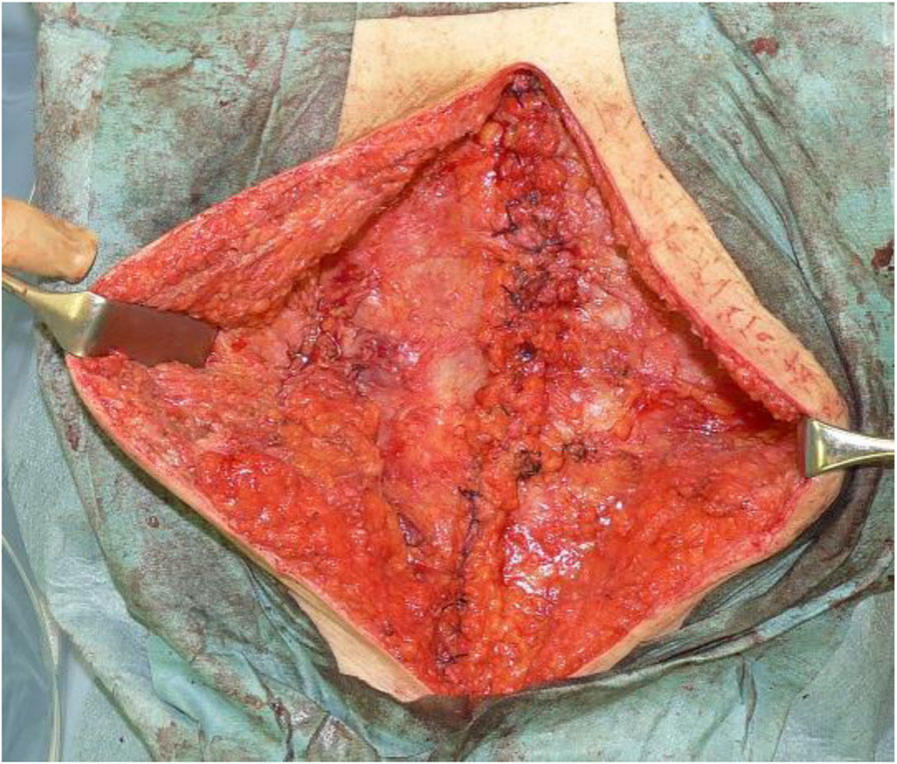
Anterior rectus sheath closure

**Fig. 6:**
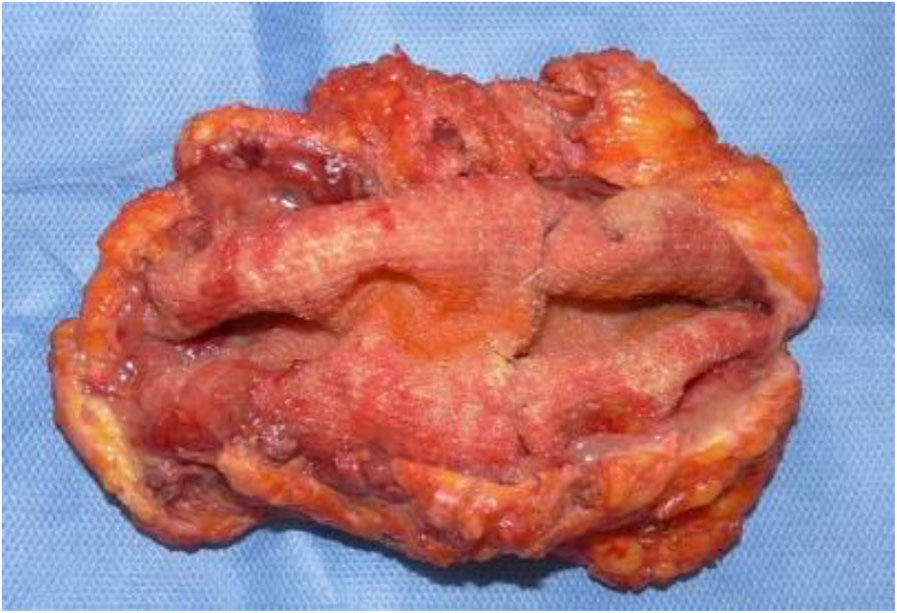
Excised abscess containing everted mesh. MRSA was identified in the abscess contents

A 66-year-old man, who underwent reconstruction of abdominal incisional hernia (8 cm in diameter) which was developed 6 months after his abdominal aorta aneurysm operation (Y-graft replacement) by retroperitoneal 15 × 8 cm Composix mesh application 10 years prior, visited our department complaining discharge from his operative wound scar. At first, conservative therapy was initiated since the discharge was serous and no symptom of infection was observed. Despite intermittent lavage and drainage for 6 months, however, the discharge was not reduced, and gradually, the properties of it changed into infective. Mesh infection was strongly suggested, and finally, we planned removal of the infected mesh. At admission, his height was 165 cm, body weight was 71.5 kg, and body temperature was normal. The fluid collection was palpable under his upper midline operative wound scar, and the skin fistula discharging contaminated exudates was observed in the middle of the wound (Fig. 1). Results of blood examination were within normal range except for slightly elevated CRP (1.46 mg/dl). Enhanced abdominal CT showed encapsulated 12 × 6.0 × 2.5 cm high-dense fluid collection involving microbubbles, suggesting abscess formation (Fig. 2). The operation was performed through upper midline incision including the skin fistula. Careful dissection successfully led complete removal of the abscess wall without damaging the peritoneum and abscess wall (Fig. 3). No intestinal fistula was observed through the procedure. Judging from the huge size of intramuscular defect of abdominal wall, simple primary closure seemed impossible whereas mesh reattachment was not preferable due to potential remnant infection. Therefore, we decided to apply posterior components separation technique (see schema in Fig. 4), and abdominal wall reconstruction was successfully achieved by anterior rectus sheath closure (Fig. 5). Resected specimen contained an everted mesh, and methicillin-resistant *Staphylococcus aureus* (MRSA) was identified in the abscess contents by postoperative microbial cultivation (Fig. 6). He discharged without any significant complications, and no recurrence of hernia and symptom of infection were observed at least 3 years after the reconstruction.

## Discussion

Incidence of mesh infection after reconstruction of abdominal incisional hernia is known to be higher than that of inguinal hernia. Although several cases of acute-onset mesh infection after abdominal incisional hernia reconstruction were reported, very few reports can be found concerning late-onset (here, we defined "late" as above 6 months after previous hernia repair) mesh infection. Only three cases were found to be reported including descriptions of the details of the case progress [2–4]. In 2006, Jezupovs et al. reported, in their retrospective analysis of 375 patients applying polypropylene mesh, a case of polyfilament mesh infection developed 18 months after recurrent incisional hernia repair followed by right subtribe laparotomy [2]. After 1-month unsuccessful abscess drainage, the 77-year-old man underwent partial removal of the *Citrobacter koseri*-infected mesh. No description was found how the reconstruction was performed in the report. In the same year, Bliziotis et al. presented a 59-year-old female case of late-onset mesh infection developed 6 months after hypogastric abdominal hernia repair followed by an ovarian cancer operation 2 years prior [3]. *S. aureus* was identified in his abscess contents. Initial 7 days antibiotic management failed, and removal of mesh combined with 2-week antibiotic medication led to the cure of infection. Unfortunately, details of the reconstruction were either not described. In 2016, Mohamed et al. reported a 44-year-old woman case of huge seroma formation diagnosed 5 years after ventral incisional hernia repair [4]. She underwent an open Roux-en-Y gastric bypass surgery and umbilical hernia repair 7 years prior, and a ventral incisional hernia repair using composite mesh 2 years later. Five months of conservative management led to evidence of highly resistant *Pseudomonas aeruginosa* infection, and she underwent mesh explantation and definitive repair with complex abdominal reconstruction combined with macroporous monofilament synthetic mesh and porcine dermal graft.

These rare cases may propose us at least two important points of discussion concerning delayed mesh infection after ventral hernia repair. First is that what causes delayed mesh infection after abdominal incisional hernia repair. At least three mechanisms can be supposed as follows: (i) remnant infection related to previous operation accompanying with contamination (which might be in association with bacterial biofilm formation on mesh), (ii) bacterial translocation followed by some kind of septic events, and (iii) de novo infection, either by mesh-intestine fistula formation or by transcutaneous. Given the four case reviews, the main cause of delayed mesh infection seems retrograde transcutaneous infection arising from prolonged drainage, accompanying bacterial biofilm, by two facts. One is that all four previous operations were performed in clean, or at least in semi-clean, conditions, and no septic event was observed in their past medical history. The other is that the types of cultured bacteria (*C. koseri*, *P. aeruginosa*, and *S. aureus*) are known to produce rich biofilm and to cause secondary remnant infection. Another point of discussion is how to manage delayed mesh infection after ventral hernia repair. It is possible to recommend avoiding prolonged conservative treatments including lavage and drainage when highly biofilm-associated bacteria were identified from the seroma/abscess contents. Additionally, as all four infected meshes were removed, mesh removal seems essential to treat delayed mesh infection in terms of biofilm debridement. However, the decision of mesh removal may often be hesitated by technical reasons especially when primary closure seems difficult or impossible due to huge intramuscular defect after mesh removal. Components separation technique application, which is described in the present case, can be of great help of designing reconstruction after removal of infected mesh for such cases. This technique is first introduced in 1990 by Ramirez et al. [5], of which the principle is basically bridging the fascial gap by separating fascial and muscular layers without using prosthetic meshes. Defects up to 20 cm in diameter can be reconstructed in a “tension-free” condition maintaining physiological abdominal wall function. Although recurrence rate is relatively high compared to mesh repair, this technique is still attractive especially in contaminated cases when mesh application should be avoided.

## Conclusions

Here, we report a very rare case of delayed MRSA-related mesh infection developed 10 years after abdominal incisional hernia repair, which was successfully treated by mesh removal and reconstruction with posterior components separation technique. An early decision of excising contaminated mesh would be preferable to extensive conservative treatments, and components separation technique can be a strong option when primary closure is not applicable for reconstruction due to a huge defect after mesh removal.

## Data Availability

There is no available data and materials to be shared.

## Abbreviations

CRP: C-reactive protein
CT: Computed tomography
MRSA: Methicillin-resistant *Staphylococcus aureus*
